# Development of a Custom-Designed, Pan Genomic DNA Microarray to Characterize Strain-Level Diversity among *Cronobacter* spp.

**DOI:** 10.3389/fped.2015.00036

**Published:** 2015-04-30

**Authors:** Ben Davies Tall, Jayanthi Gangiredla, Gopal R. Gopinath, Qiongqiong Yan, Hannah R. Chase, Boram Lee, Seongeun Hwang, Larisa Trach, Eunbi Park, YeonJoo Yoo, TaeJung Chung, Scott A. Jackson, Isha R. Patel, Venugopal Sathyamoorthy, Monica Pava-Ripoll, Michael L. Kotewicz, Laurenda Carter, Carol Iversen, Franco Pagotto, Roger Stephan, Angelika Lehner, Séamus Fanning, Christopher J. Grim

**Affiliations:** ^1^Center of Food Safety and Applied Nutrition, U. S. Food and Drug Administration, Laurel, MD, USA; ^2^UCD Centre for Food Safety, School of Public Health, Physiotherapy and Population Science, University College Dublin, Dublin, Ireland; ^3^WHO Collaborating Centre for Cronobacter, Dublin, Ireland; ^4^Center of Food Safety and Applied Nutrition, U. S. Food and Drug Administration, College Park, MD, USA; ^5^College of Life Sciences, University of Dundee, Dundee, UK; ^6^Food Directorate, Bureau of Microbial Hazards/Health Canada, Ottawa, ON, Canada; ^7^Institute for Food Safety and Hygiene, University of Zurich, Zurich, Switzerland

**Keywords:** *Cronobacter*, microarray, genomic diversity, pan genomic, phylogenic divergence

## Abstract

*Cronobacter* species cause infections in all age groups; however neonates are at highest risk and remain the most susceptible age group for life-threatening invasive disease. The genus contains seven species:*Cronobacter sakazakii*, *Cronobacter malonaticus*, *Cronobacter turicensis, Cronobacter muytjensii*, *Cronobacter dublinensis*, *Cronobacter universalis*, and *Cronobacter condimenti*. Despite an abundance of published genomes of these species, genomics-based epidemiology of the genus is not well established. The gene content of a diverse group of 126 unique *Cronobacter* and taxonomically related isolates was determined using a pan genomic-based DNA microarray as a genotyping tool and as a means to identify outbreak isolates for food safety, environmental, and clinical surveillance purposes. The microarray constitutes 19,287 independent genes representing 15 *Cronobacter* genomes and 18 plasmids and 2,371 virulence factor genes of phylogenetically related Gram-negative bacteria. The *Cronobacter* microarray was able to distinguish the seven *Cronobacter* species from one another and from non-*Cronobacter* species; and within each species, strains grouped into distinct clusters based on their genomic diversity. These results also support the phylogenic divergence of the genus and clearly highlight the genomic diversity among each member of the genus. The current study establishes a powerful platform for further genomics research of this diverse genus, an important prerequisite toward the development of future countermeasures against this foodborne pathogen in the food safety and clinical arenas.

## Introduction

*Cronobacter* is an opportunistic food borne pathogen. The genus contains seven species: *Cronobacter sakazakii*, *Cronobacter malonaticus*, *Cronobacter turicensis, Cronobacter muytjensii*, *Cronobacter dublinensis*, *Cronobacter universalis*, and *Cronobacter condimenti*; and is capable of causing illness among all age groups of the population (1). Population groups especially at risk of severe infections include neonates (infants less than 4 weeks of age) and infants, as well as elderly and immuno-compromised adults. Infantile disease presents clinically as meningitis, septicemia, and necrotizing enterocolitis and its occurrence being epidemiologically linked to the consumption of temperature-abused, reconstituted, intrinsically or extrinsically contaminated powdered infant formula (PIF) (2–4). Infections such as pneumonia, septicemia, catheter-associated, and urinary tract infections in adults have also been reported and the epidemiology of these cases suggests that other potential sources of illness exist (1). Surveillance studies have shown that *Cronobacter* contaminate a multitude of foods and environments, including water, infant foods (PIF, follow-up formula), dried milk protein products, cheese, licorice, candies, dried spices, teas, nuts, herbs, filth and stable flies, and PIF or milk powder production facilities and household environments (4, 5).

The Food and Drug Administration’s (FDA) and its food safety partners’ abilities to protect the global food supply and public health, at large, is augmented through the development of methods that can rapidly detect and characterize foodborne pathogens. The goals of this study were to characterize 126 *Cronobacter* and related strains for their genomic content and describe the phylogenetic diversity while also developing a novel next generation sequence-based, custom-designed pan genomic microarray platform as a highly discriminatory characterization and identification tool for public health and source attribution. The microarray was developed during 2009–2013 through the leveraging of next generation, whole genome sequencing efforts of a five-member International *Cronobacter* Consortium (ICC). This report provides the description of a single lab validation study which took place during April, 2013 to October, 2014 using strains, representative of the seven *Cronobacter* species and strains from phylogenetically related genera.

By applying highly discriminatory sequence-based molecular methods, such as DNA microarray and its concise annotation, species identity and phylogenetic relatedness among strains isolated during surveillance and outbreak investigations can be better understood. With this broader understanding, food safety and public health groups can quickly and accurately identify organisms responsible for infections so that patients can be given rapid and appropriate treatment and the associated contaminated food products can be removed from commerce; thereby helping to ensure the safety and reliability of the food supply.

## Materials and Methods

### Bacterial strains

The strains evaluated in this study consisted of 49 *C. sakazakii*, 27 *C. malonaticus*, 12 *C. muytjensii*, 8 *C. turicensis*, 19 *C. dublinensis*, 2 *C. universalis*, and the single known strain of *C. condimenti* from the combined FDA-ICC laboratory culture collection and the strains, their metadata are listed in Table S1 in Supplementary Material. These 118 strains represent isolates obtained from clinical, food, and environmental sources and from diverse geographical locations. Eight phylogenetically related species representative of *Salmonella enterica* Typhimurium, *Klebsiella pneumoniae*, *Citrobacter freundii*, *Siccibacter turicensis* (one strain), *Franconibacter helveticus* (two strains), and *Franconibacter pulveris* (two strains) were also included. Assignment of *Cronobacter* species identification to these strains was performed according to the proposed classification scheme as suggested by Iversen et al. (6) and Joseph et al. (7) using a Gram-negative card analyzed with the Vitek-2 Compact instrument (Biomerieux, Hazelwood, MO, USA) and its version 5.03 software which identifies *Cronobacter* species using a slash-line protocol similar to what is used for the identification of *Escherichia coli*, *Salmonella* spp., and other enteric Gram-negative bacterial pathogens. All of the *Cronobacter* strains were identified by Vitek-2 Compact analysis as members of the *Cronobacter* genus complex, and except for *C. condimenti*, all were PCR-positive for the 350-bp amplified region of the zinc metalloprotease (*zpx*) gene, a genus-specific target previously reported by our group (8) prior to the isolation and description of *C. condimenti*. It was shown later by Stephan et al. (9) that *C. condimenti* possesses a *zpx* homolog. The species identity of the isolates was also confirmed using the species-specific *rpoB* PCR assays as described by Stoop et al. (10) and Lehner et al. (11) and the *cgcA* species-specific PCR assay as described by Carter et al. (12). Additionally, this collection of isolates was subjected to RepF1B plasmidotyping (13) and molecular serogrouping (14–17), the results of which further corroborated results of the *rpoB*- and *cgcA*-based PCR species identification assays for each strain [data reported previously in Ref. (13–17)] and provides an accurate assessment of the validity of the microarray’s ability to identify unknown organisms as *Cronobacter*.

### Bacterial genomic preparations

Strains were grown overnight at 37°C in 5 ml of Trypticase soy broth (BBL, Becton Dickinson, Franklin Lakes, NJ, USA) supplemented with 1% NaCl (final conc.), shaking at 160 rpm. Genomic DNA was isolated from 2 ml of the culture using a robotic QIAcube workstation with its automated Qiagen DNeasy chemistry (Qiagen, Germantown, MD, USA) following the manufacturer’s recommendations. Typically, 5–15 μg of purified genomic DNA was recovered in a final elution volume of 200 μl. The purified DNA was further concentrated using an Amicron Ultracel-30 membrane filter (30,000 molecular weight cutoff, 0.5 ml, Millipore Corp. Billerica, MA, USA) to a final volume of approximately 10–25 μl.

### Microarray design

The microarray used in this study is an Affymetrix MyGeneChip Custom Array (Affymetrix design number: FDACRONOa520845F) and was developed utilizing the whole genome sequences of 15 *Cronobacter* strains, which also possessed 18 plasmids (Table 1). We previously reported a pan-genome analysis of the *Cronobacter* genus, utilizing eight of these reported genomes, which covered the contemporary species and subspecies (18). We leveraged the results of this previous report and expanded the scope of the core/pan-genome analysis to include more strains of the predominant clinical species, *C*. *sakazakii* and *C*. *malonaticus*, and the newly described species, *C*. *condimenti*. Genomes (and NCBI GenBank accession numbers) used in the design of the chip included *C. sakazakii* ATCC BAA-894 (chromosome, NC_009778.1; plasmid pESA3, NC_009780.1; and plasmid pESA2, NC_009779.1), *C. sakazakii* ES713 (4.01C) (chromosome and pESA3-like plasmid, AJLB00000000.1), *C. sakazakii* 2151 (chromosome, pESA3-like plasmid, and plasmid pCSA2151, AJKT01000000.1), *C. sakazakii* ES35 (chromosome, pESA3-like plasmid, and pCTU3-like plasmid; AJLC00000000.1), *C. sakazakii* ES15 (chromosome, NC_017933.1), *C. sakazakii* E764 (chromosome and pESA3-like plasmid; AJLA00000000.1), *C. dublinensis* ssp. *lactaridi* LMG23825^T^ (chromosome and pEAS-3/pCTU1-like plasmid, AJKX01000000.1), *C. dublinensis* ssp. *dublinensis* LMG23823^T^ (chromosome and pEAS-3/pCTU1-like plasmid, AJKZ01000000.1), *C. dublinensis* ssp. *lausannensis* LMG23824^T^ (chromosome and pEAS-3/pCTU1-like plasmid, AJKY01000000.1), and *C. muytjensii* strain 51329^T^ (chromosome, AJKU01000000.1). *C. turicensis* strain LMG23827^T^ (chromosome, NC_013282.2; and plasmids pCTU1, NC_013283.1; pCTU2, NC_013284.1; pCTU3, NC_013285.1), *C. universalis* strain NCTC9529^T^ (formerly designated as *Cronobacter* genomosp. Group 1, chromosome and pEAS-3/pCTU1-like plasmid, AJKW01000000.1), *C. condimenti* strain LMG26250^T^ (chromosome and pEAS-3/pCTU1-like plasmid, CAKW00000000.1), *C. malonaticus* strains LMG23826^T^ (chromosome and pEAS-3/pCTU1-like plasmid, AJKV010000 00.1), and *C. malonaticus* ENBT0334 (FDA827; chromosome and pEAS-3/pCTU1-like plasmid, JXTD00000000.1).

**Table 1:** **Number of gene features identified in 13 of the 18 strains that was used to develop the microarray**.

Species and strain	Number of gene alleles from each strain	Number present with array (%)^a^	Expected total gene features	Total number of gene features present in the array^a^
*C. sakazakii*				
ATCC BAA-894 @5 μg	2,035	1,904 (93.5)	3,954	3,875
ATCC BAA-894 @2 μg	2,035	1,819 (89.4)	3,954	3,152
ATCC BAA-894 @10 μg	2,035	1,782 (87.5)	3,954	3,654
4.01C	139	132 (94.9)		3,465
2151	201	171 (85.0)		3,372
ES35	202	188 (93.0)		4,172
Es764	304	266 (87.5)		3,311
*C. turicensis* LMG23827^T^	4,402	4,039 (91.7)	4,470	4,581
*C. malonaticus*				
LMG23826^T^	1,582	1,434 (90.6)	3,593	3,693
CDC2193-01	257			
*C. dublinensis*				
*dublinensis* LMG23823^T^	781	745 (95.4)	4,165	3,868
*lausannensis* LMG23824^T^	2,580	2,386 (92.5)	3,905	3,972
*C. muytjensii* ATCC 51329^T^	1,754	1,708 (97.3)	3,633	3,099
*C. universalis* NCTC9529^T^	1,315	1,201 (91.3)	3,720	3,477
*C. condimenti* LMG26250^T^	2,611	2,498 (95.7)	3,750	3,613

*^a^Totals were calculated using only hybridization reactions (MAS5 table) that contained 5 μg concentrations of DNA, except where noted*.

**Figure 1:**
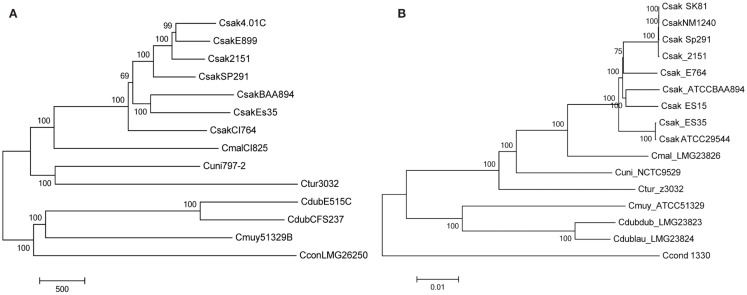
Molecular phylogenetic analysis representing the evolutionary relationship of *Cronobacter* strains used to develop the microarray **(A)**, after interrogation of the strains with the *Cronobacter* microarray and applying the RMA-derived presence/absence gene algorithm. The microarray experimental protocol as described by Jackson et al. (20) was used for the interrogation of the strains and for the analysis. The tree was generated using the neighbor-joining method using MEGA6 (21) and 1000 bootstrap replicates; the scale bar indicates number of presence/absence gene differences. For comparison, a phylogenetic tree derived from whole-genome SNP analysis is shown **(B)**. The SNP-based evolutionary history was also inferred using the neighbor-joining method using MEGA6 (21) and 1000 bootstrap replicates; the scale bar indicates 0.01 base differences per sequence.

For both the core genome which comprised an estimated 3,036 genes, and the dispensable genome (genes present in two or more strains) or “unique genes,” specific to single strains, the microarray was designed such that gene homologs across multiple species were represented by the smallest number of probe sets that theoretically covered all alleles of the gene target. Due to its phylogenetic placement within the genus (See Figure 1A) and being a closed genome, the core genome that was chosen to represent the genus *Cronobacter* was that of *C. turicensis* LMG23827^T^ (z3032) (18). We used a ≥97% identity threshold level between the alleles of *C. turicensis* and gene homologs of the other sequenced strains to positively predict allelic coverage. For homologs of the core genome from other species that diverged below the identity threshold, those alleles were also included on the microarray. For the *C*. *dublinensis* group, the core genome homologs from *C*. *dublinensis* ssp. *lausannensis* LMG23824^T^ were preferentially used based on average nucleotide identity across the whole genome, unless coverage was below the identity threshold. For dispensable genomic regions and mobilome genes, gene homologs were assessed as above to determine the smallest number of alleles for inclusion in the microarray design. In total, 19,287 *Cronobacter* gene targets were captured on the microarray.

To determine if new gene features from other phylogenetically related organisms such as *Citrobacter*, *Klebsiella*, *Salmonella*, and *E. coli* may have been acquired through horizontal gene transfer mechanisms (i.e., transformation, conjugation, or transduction events; during adaptation to new environments), an additional 2,371 virulence factor gene sequences of these phylogenetically related Gram-negative bacteria, obtained from the MvirDB virulence database housed at the Lawrence Livermore National Laboratory (19), were also included in the microarray design.

Each gene is represented on the array by 22 unique 25-mer oligonucleotide probes, as described by Jackson et al. (20), consisting of a set of 11 probe pairs which contain a perfect match (PM) probe and a mismatch (MM) probe for each gene region. The PM probe matches the target/reference sequence perfectly while the MM probe contains a single nucleotide MM in the central (13th) nucleotide position. The rationale for including the MM probe is for measuring and thus correcting for non-specific hybridization. For a small percentage of gene homolog families, more than one probe set for each allele was included for evaluation, with each probe set consisting of either unique or cross-hybridizing probes, or a mixture of both.

### Microarray hybridization

Genomic DNA was fragmented by incubating at 37°C for 1 min in a 20 μl reaction containing 1× One-Phor-All Plus Buffer (GE Healthcare) and 0.01 U DNase I (GE Healthcare) as described by Jackson et al. (20). The fragmentation was heat-inactivated at 99°C for 15 min. The fragmented DNA was 3′-end labeled by adding 4 μl of 5× terminal transferase buffer (Promega), 1 μl 1 mM biotin-11-ddATP (PerkinElmer NEL508), and 2 μl (60 U) of terminal transferase enzyme (Promega). Labeling was carried out for 4 h at 37°C followed by heat inactivation at 98°C for 1 min.

Hybridizations were performed according to the Affymetrix GeneChip Expression Analysis Technical Manual for a 49-format array (22). Briefly, each sample was added to 146 μl of the hybridization buffer [comprised 100 μl of 2× hybridization buffer, 3.3 μl of a 3 nM B2 oligonucleotide solution, 2 μl each of a 10 mg/ml Salmon DNA and 50 mg/ml BSA solutions, and 15.5 μl of DMSO (Sigma-Aldrich, Inc. St. Louis, MO, USA)] and was denatured at 98°C for 1 min. The denatured samples were added onto the Affymetrix arrays, which were then incubated at 45°C, with rotation (60 rpm) for 16 h in a hybridization oven. Following hybridization, wash and stain procedures were carried out on an Affymetrix FS-450 fluidics station using the mini_prok2v1_450 fluidics script (23). Reagents for washing and staining were prepared according to the GeneChip^®^ Expression Analysis Technical Manual (22). The following exceptions were made to the wash and stain procedure: streptavidin solution mix (vial 1) was replaced with SAPE solution mix (Life Technologies, Grand Island, NY, USA). Arrays were scanned using an Affymetrix GeneChip^®^ Scanner 3000 running AGCC software.

### Microarray data analysis

For each gene represented on the microarray, probe set intensities were summarized using the Robust MultiArray Averaging (RMA) function in the Affymetrix package of R-Bioconductor as described by Bolstad et al. (24). Briefly, RMA summarization of probe level data was done by performing three individual treatments on all of the experimental data (CEL file) in succession. First, probe specific correction of the PM probes was done using a model based on the observed intensities being the sum of signal and noise. Second, quantile normalization was performed on the corrected PM probe intensities. Finally, a median polishing algorithm was used to summarize the background-corrected, normalized probe intensities to generate a final probe set value.

### Calculating gene differences and generating dendrograms

Robust MultiArray Averaging-summarized probe set intensities were compared across all strains for each gene. If the same gene in different strains had an RMA intensity difference greater than eightfold (log_2_ = 3), then that gene was considered to be “different.” Using this criterion, a strain versus strain gene-difference matrix was generated; where the difference matrix represents the number of genes/alleles that differs between any two isolates. Gene-difference matrices were converted to dendrograms using the *hclust* function in the base package as well as the *phylo* function in the *ape* package of R-Bioconductor. Hierarchical clustering was performed with the RMA-summarized probe set intensities using the MADE4 package of R-Bioconductor. Phylogenetic trees were made using the nearest neighbor-joining method via the MEGA 5 software package as described by Jackson et al. (20). Scatter plots were used to verify with the RMA-summarized probe set intensities as described by Jackson et al. (20).

## Results

### Optimization and validation of the *cronobacter* microarray

Optimization and validation of the *Cronobacter* microarray was performed by performing hybridization experiments with the strains used to design the microarray. To determine the amount of genomic DNA to be used for microarray hybridization experiments, experiments using 2, 5, and 10 μg of purified genomic DNA isolated from *C. sakazakii* ATCC BAA-894 were conducted. These experiments revealed that 5 μg was optimal in terms of sensitivity, as hybridization experiments conducted with 5 μg identified 93.5% of gene alleles from BAA-894 present on the microarray, versus 89.4 and 87.5% for 2 and 10 μg, respectively (Table 1). The reason why the 10 μg amount failed to give equal or better results than the 5 μg amount is due to greater hybridization of the labeled DNA to the probes which led to fluorescent bleed over and increased background noise. Similar results were observed for all other strains of *Cronobacter* used in the microarray design when 5 μg of DNA was used (Table 1). Overall, the microarray identified 91% of gene alleles of the 15 genomes used to develop the microarray.

Furthermore, 5 μg of DNA of *C*. *sakazakii* ATCC BAA-894 yielded a total gene content of 3,875 (98%) genes, for which we expected 3,954 genes when taking total core and pan-genome alleles into account (Table 1). All other chip design strains yielded gene content counts close to expected values (Table 1), except for *C*. *muytjensii* ATCC 51329^T^, which only identified 85% of expected genes (3,099 of 3,633). This is most likely due to the microarray design in which certain gene alleles from *C*. *dublinensis* ssp. *lausannensis* LMG23824^T^ were used to represent not only the species, *C*. *dublinensis*, but also *C*. *muytjensii*. In a number of cases, the sequence divergence was too great and the probes from *C*. *dublinensis* ssp. *lausannensis* LMG23824^T^ failed to identify gene homologs in *C*. *muytjensii* ATCC 51329^T^. This is also true for *C. universalis* NCTC9529^T^ and *C. condimenti* LMG26250^T^, for which alleles of *C*. *turicensis* z3032 were used to represent core genes. Even though the results were not as expected and a complete explanation was not readily apparent, the *Cronobacter* microarray, none the less, was very capable of separating strains of each species into the appropriate taxa based on the alleles captured by the microarray design. This is an area of improvement in the next microarray design, which is currently underway. Furthermore the next array design will contain more genomic information from the sequencing of a greater number of strains so that greater genomic content and diversity will be captured. For some microarray design strains, the gene content revealed by the microarray hybridization was higher than expected, due to cross-hybridization with multiple alleles with a gene homolog family (Table 1).

Another set of data which validate the pan genomic approach to the development of this microarray is shown in Figure 1. Two phylogenetic trees generated by the analysis of microarray presence/absence calls from the interrogation 14 of the 15 strains used to develop the microarray (Figure 1A) is shown in comparison to a tree used the whole genome sequences of these same strains (Figure 1B). In both instances, the analyses gave identical sets of data showing that the two phylogenetic trees recapitulated each other and also demonstrate the species-delineated bidirectional evolutionary nature of members of the genus *Cronobacter* as reported by Grim et al. (18) and Stephan et al. (25). Each tree shows the divergence of *C. sakazakii*, *C. malonaticus*, *C. universalis* and *C. turicensis* strains from *C. muytjensii*, and *C. dublinensis*. These analyses also presented *C. condimenti* as a distinct outlier.

The probe set design utilized in this pan genomic genotyping array also allowed for a highly accurate determination of gene allele divergence for these 126 isolates (Table S1 in Supplementary Material). Another way of assessing how well different strains are related to one another is by using Pearson’s correlation or Pearson Product Moment Correlation (PPMC) which shows the linear relationship between two strains. Table S3 in Supplementary Material shows the results of this analysis and demonstrates specificity of the clustering of each of the species groups in terms of a percentage. For example, comparison of the percentages of the 19 *C. dublinensis* strains to those of *C. condimenti* shows that these two species differ from one another by 60–70%.

### Species identification and subtyping using the *cronobacter* microarray

**Figure 2:**
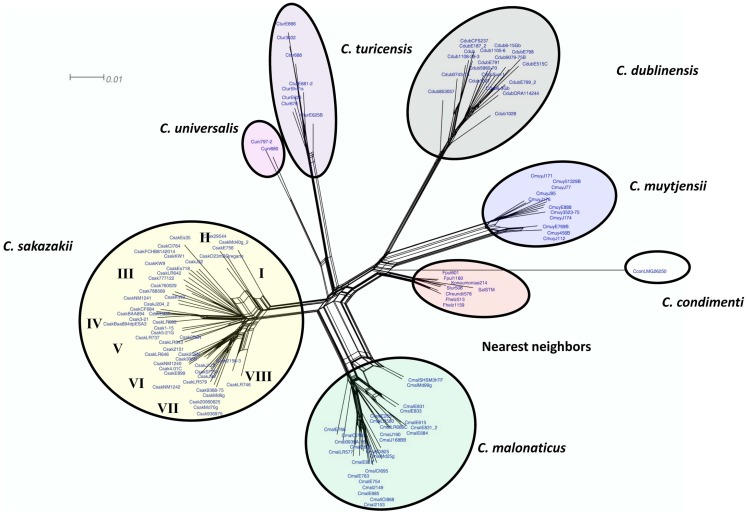
**Neighbor net (SplitsTree4) analysis of 126 *Cronobacter* and phylogenetically related strains, which were generated from the gene-difference matrix shown in Table S1 in Supplementary Material**. The microarray experimental protocol as described by Jackson et al. (20) was used for the interrogation of the strains and for the analysis. The phylogenetic tree illustrates that the *Cronobacter* microarray could clearly separate the seven species of *Cronobacter*, with each species forming their own distinct cluster. The tree was generated using SplitsTree4 neighbor net (33). *C. sakazakii* subclades are denoted as Roman numerals I–VII. The scale bar represents a 0.01 base substitution per site.

To assess and demonstrate the ability of the *Cronobacter* microarray to identify strains to the correct species and subspecies taxa, hybridization experiments with 118 strains of *Cronobacter* were performed (Table S1 in Supplementary Material). Additionally, eight strains from taxonomically related species were also analyzed (Table S1 in Supplementary Material). After applying the RMA-derived presence/absence gene algorithm, a gene-difference matrix was generated from the interrogation of the 125 strains (Table S2 in Supplementary Material). From this data, a phylogenetic tree was generated, which demonstrates that the microarray could correctly identify all strains to the species and subspecies taxonomic groups within *Cronobacter* (Figure 2). Within each species cluster, further delineation was seen. For example, the *C. sakazakii* and *C. malonaticus* species groups comprised eight and five main clades each having numerous subclades. The *C. muytjensii* and *C. turicensis* clades comprised two main subclades and the *C. dublinensis* cluster comprised four clades. The two *C. universalis* strains formed a single cluster and the lone *C. condimenti* strain was a distant outlier. It is important to note that an absolute correlation was found between the identities of each strain obtained by the validated end-point PCR species-specific *rpoB* and *cgcA* PCR assays and the species identities obtained by using the microarray (10–12). Furthermore, these results support the divergence of the genus from the most recent hypothetical ancestral species into two main clusters, one consisting of *C. dublinensis* and *C. muytjensii* and the other comprised *C. sakazakii*, *C. malonaticus*, *C. universalis*, and *C. turicensis* as described by Grim et al. (18). As shown in this study, *C. condimenti* was a distant outlier of these two main phylogenetic groups, but is more closely related to the *C. dublinensis–C. muytjensii* cluster and supports this proposition as advanced by Joseph et al. (7).

### Genomic diversity among *cronobacter* spp. Revealed by the *cronobacter* microarray

Among the many features of the *Cronobacter* microarray, the most powerful is the ability of the microarray to assess the dispensable genome within and between species; further elucidating evolutionary relationships among disproportionately distributed, but vertically acquired genomic features, as well as horizontally acquired mobile elements. Four case scenarios are used to demonstrate how the microarray can be used to understand the genomic diversity of *Cronobacter*.

The first case scenario demonstrates how the microarray can be used to identify *C. dublinensis* strains to the subspecies level. Iversen et al. (6) described two phenotypic traits that could be used to separate the three subspecies of *C. dublinensis*; namely, *C. dublinensis* spp. *dublinensis* is able to utilize both malonate and *myo*-inositol, while *C. dublinensis* ssp. *lactaridi* utilizes only *myo*-inositol and *C. dublinensis* ssp. *lausannensis* cannot utilize either substrate. The gene alleles represented on the microarray for malonate utilization are found in Genome Region (GR) GR34 as described by Grim et al. (18), GR34 encodes for the components of enzymes and proteins involved in the decarboxylation of malonate by *C*. *dublinensis* subspecies i.e., C. *dublinensis* ssp. *dublinensis* LMG23823^T^. These include a malonate transporter gene, *mdcF*, and *mdcE*, which encodes for a stabilization protein, which is thought to stabilize MdcF with the beta chain of the acetyl-coenzyme-A carboxyl transferase. Malonate decarboxylase comprises a delta and alpha subunit and is encoded by two genes, *mdcAC*. Finally, a gene encoding for a 2-(5″-triphosphoribosyl)-3′-dephosphocoenzyme-A synthase, which is also thought to stabilize the coenzyme-A complex so that the proper conformation of MdcAC is maintained in a high substrate affinity configuration, is also part of this gene cluster. Hybridization of the type species strain *C*. *dublinensis* ssp. *dublinensis* LMG23823^T^ (synonym, CFS237) showed that this strain possessed genes associated with all of the above gene targets. Comparison of hybridization results among 18 other C. *dublinensis* strains isolated from various clinical, food, and environmental sources revealed that the malonate operon is indeed unique to the five strains of ssp *dublinensis* (Table S4 in Supplementary Material).

Catabolism of the substrate, *myo*-inositol, is encoded in an operon consisting of 10 genes in *Cronobacter* (26). The gene alleles that represent the gene cluster for *myo*-inositol utilization [GR29 from Grim et al. (18)] within strains of *C*. *dublinensis* is from ssp. *lactaridi* strain LMG 23825^T^. Also shown in Table S4 in Supplementary Material are results of the hybridization reactions with the *C. dublinensis* strains and these *myo*-inositol utilization genes. The five C. *dublinensis* ssp. *dublinensis* isolates were found to possess this operon, as were the eight isolates of *C. dublinensis* ssp. *lactaridi*. Conversely, microarray analysis of the six other *C. dublinensis* strains, 6_15Gb, 9079_75, 515C (synonymous with LMG 23824^T^), E798, E799_2, and ORA114244, revealed that these strains did not possess either the *myo*-inositol or the malonate catabolic gene clusters, thus confirming their identities as *C. dublinensis* ssp. *lausannensis*.

In addition to *C. dublinensis* ssp. *dublinensis* being able to utilize malonate, Iversen et al. (6) described malonate utilization by other *Cronobacter* species such as *C. malonaticus*, *C. muytjensii*, *C. turicensis*, and *C. universalis*. Furthermore, Joseph et al. (7) reported that *C. condimenti* can also utilize malonate. The *Cronobacter* microarray contains species-specific gene alleles of each annotated gene of the malonate operon (Table S5 in Supplementary Material). In this regard, the microarray captured species-specific gene divergence for these shared pan-genomic features, and further used that information to genotype these strains. The microarray can also reveal nucleotide identity divergence among strains of the same species. For example, hybridization of 27 *C. malonaticus* strains showed that all of the strains possessed the entire gene cluster of *C. malonaticus* LMG 23825^T^ (synonymous with CI825) (Table S5 in Supplementary Material). A similar level of homology was observed among strains of *C*. *muytjensii*, but not for *C. universalis, C. condimenti*, and *C. turicensis*, suggesting that different levels of sequence divergence do exist among species. None of the 49 *C. sakazakii* strains hybridized with any of these probes supporting the phenotypic evidence described by Iversen et al. (6) that *C. sakazakii* is malonate negative. However, Iversen et al. (6) do mention that some *C. sakazakii* strains may utilize malonate, so more strains need to be evaluated.

In addition to these two discriminatory phenotypic traits, Grim et al. (18) identified several genomic features that could be used in a molecular-based dichotomous key to determine subspecies identity within *C*. *dublinensis*. However, the authors only analyzed the type strains of each subspecies, so it is unclear whether the distribution of genomic features holds true across more strains. By expanding our interrogation of the species to 19 isolates, we found that many of the genomic regions identified are indeed discriminatory, to include GR2 (taurine metabolism) and 70, missing in ssp. *dublinensis*; GR15 and 16, missing in ssp. *lausannensis*; GR35 and 85, present only in ssp. *dublinensis;* and GR66, present in only ssp. *lausannensis*. We also found that some genomic features that appeared to be discriminatory by Grim et al. (18) may not be, due to strain-to-strain differences within subspecies as captured by the *Cronobacter* microarray. For example, genes that encode β-fimbriae were present in the type strain of *C*. *dublinensis* ssp. *lactaridi*; however, they were also present in three other strains, which were all *C*. *dublinensis* ssp. *lausannensis*.

In a second example, the *Cronobacter* microarray used to examine the distribution of dispensable fimbriae-encoding operons within individual species. There are 179 gene alleles representing eight fimbriae gene clusters on the microarray from Grim et al. (18): GR9, encoding π-fimbriae; GR76, encoding β-fimbriae; GR6 and GR52, encoding γ-4 fimbriae from; GR82 and GR126 encoding γ-1 fimbriae, GR112, encoding κ-fimbriae, and GR55, encoding curli fimbriae. Again, we can use the microarray interrogation of these strains of *Cronobacter* to attain a better assessment as to the distribution of these appendage-encoding operons. For example, Grim et al. (18) reported that the genes encoding for the π-fimbriae were absent in one genome, that of *C*. *sakazakii* BAA-894. The *Cronobacter* microarray revealed that this genomic feature was present in two of five isolates of *C*. *dublinensis* ssp. *dublinensis*, three of six strains of *C*. *dublinensis* ssp. *lausannensis*, all strains of *C*. *muytjensii* and *C*. *malonaticus*, and only three of eight strains of *C*. *turicensis*, and 38 strains of *C*. *sakazakii*, as well as present in the single strain of *C. condimenti*. So, four of the seven species demonstrate a narrower range of distribution of this operon. This is a trend seen with several of the other fimbriae gene clusters. GR126 was found to be only present in nine strains of *C*. *sakazakii*, and GR6 was only present in 15 of 19 strains of *C*. *dublinensis* and four of eight strains of *C*. *turicensis*. Conversely, some fimbriae operons demonstrated an expanded range of distribution as compared to that reported by Grim et al. (18). For example, GR 82 (encoding for γ_1_ fimbriae) was present in three strains of *C*. *dublinensis* ssp. *dublinensis* in addition to all strains of *C*. *sakazakii* and *C*. *malonaticus*, and β-fimbriae (GR76) were present in three strains of *C*. *dublinensis* ssp. *lausannensis*, 6-15, E799 and ORA114244, in addition to the type strain of *C*. *dublinensis* ssp. *lactaridi* and all strains of *C*. *sakazakii*, GR52 (encoding for γ_4_ fimbriae) was found in 32 strains of *C*. *sakazakii*, in addition to seven of eight *C*. *turicensis* and 10 of 19 *C*. *dublinensis* strains. Species-specific alleles of the curli gene cluster (GR55) were present in all strains of *C*. *malonaticus*, *C*. *dublinensis* ssp. *dublinensis*, and *C*. *universalis*, but only three of eight strains of *C*. *turicensis* and additionally one strain of *C*. *dublinensis* ssp. *lactaridi*.

The third case scenario involves assessing the genomic diversity among 12 *C. sakazakii* which are representative of the eight subclades (I–VIII) shown in Figure 2. The microarray can be used to describe the total gene content of any set of organisms. This is useful in understanding gene features which may contribute to a strain’s divergence. The distribution and prevalence of such gene features are shown in Table S6 in Supplementary Material. Several interesting genomic topographies were found. For example, there were 3937 phage-related alleles represented on the microarray. Among the 12 highlighted strains from Figure 2, two phage gene clusters which appeared to be from *C. condimenti* were present in two of these *C. sakazakii* strains. Also two of these strains possessed another phage gene cluster that seems to be related to a phage present in *C. dublinensis*. There seems to be at least six phage gene clusters or gene remnants from *C. malonaticus* represented as well in these strains. One other clinical strain 20060625 possessed the entire phage gene cluster that was also seen in *C. sakazakii* strain 2151. There were remnants of other phage gene clusters being present from *C. sakazakii* strains 713 and BAA-894. In *C. sakazakii* strain 3–21, there was an entire phage cluster from BAA-894 (1844.1425-1508) that was associated with a core gene cluster containing an *ompW* and indole 3-glycerol phosphate synthase genes. Clinical strain Es35 also possessed much of this phage gene cluster. However, there was a phage gene cluster (1844.2133-0.2149) that was absent in these strains, but present in strain BAA-894. There were multiple type six secretion systems dotted through the *C. sakazakii* genomic topography, with one being contiguous with a phage gene cluster from strain BAA-894. Several core gene regions encoding genes for a ferric hydroxamate outer membrane receptor FhuA, outer membrane pore protein E precursor gene, chitinase, vitamin B12 ABC transporter were seen in these twelve strains. Lastly, whole copies of pCTU3 from *C. turicensis* were seen in four of these 12 strains. Phage remnants from *C. turicensis* were also dotted throughout the genomic topography of these strains.

The total number of gene features present among these *C. sakazakii* strains ranged from 2602 genes in the domestic house fly, *Musca domestica* gut isolate which clustered in subclade I to 4177 gene features present in the clinical strain Es35 which clustered in subclade III and these results are shown in Table S6 in Supplementary Material. On average, food-associated isolates (E756, 777122, J2904, KW9, and 3–21) possessed 3529 microarray gene features, while clinical isolates (ES35, 2151, 306N, 206N, 20060625, and 2156) possessed 3299 microarray gene features suggesting that there may be a trend in human-host associated strains for a reduction in genome features, or pathoadaption as suggested by Yan et al. (27) compared to food-associated strains, but this trend was found to be not significant (two-tailed student *t*-test, *p* value of 0.50). Furthermore, to answer this question more definitely, a greater number of strains need to be evaluated.

The last case scenario involves using the microarray to understand the distribution and prevalence of a particular gene and in this case a gene encoding for a putative virulence factor, the macrophage infectivity potentiator-related protein which is thought to play a role in survival of *Cronobacter* within macrophages as the organism transitions itself from its systemic lifestyle through its tropism-like route to the blood–brain barrier. This gene feature was described in the genome studies by Grim et al. (18) and Kucerova et al. (28) and the gene encoding for the macrophage infectivity potentiator-related protein was found to be present in *C. turicensis*, *C. malonaticus*, *C. muytjensii*, and *C. sakazakii* (Table S7 in Supplementary Material). Microarray analysis confirmed that *C. turicensis*, *C. malonaticus*, *C. muytjensii*, and *C. sakazakii* do possess the gene and that *C. dublinensis*, *C. universalis* and *C. condimenti* do not possess the gene. Also microarray analysis demonstrated that the alleles for the macrophage infectivity potentiator-related protein gene have diverged to the extent that each has become a species-specific trait.

### Genes identified in *cronobacter* species which were associated with gene features from the MvirDB database

Using the probe sets designed from the MvirDB database an attempt was made to see if any of the *Cronobacter* species possessed genes encoding for common virulence factors from other phylogenetically related enteric pathogens. There were 317 virulence factor genes identified through microarray analysis and these are summarized in Table S8 in Supplementary Material. Interestingly, several genes involving iron and transport of iron were found (i.e., fused ferrous iron transporter, cytoplasmic ferritin, and a gene encoding for a dihydrofolate reductase), also some phage associated genes were found (i.e., a gene encoding a CP4-6 prophage; predicted DNA repair protein, and an integrase of *Shigella flexneri* bacteriophage X), a homolog of an ES-beta-lactamase gene was found in 35/126 isolates (27%), and lastly a gene encoding for a putative type IV pilus operon lipoprotein was identified to name a few genes.

## Discussion

Two previous microarray studies have been used to understand the genomic diversity within *Cronobacter*. The first study, described by Healy et al. (29), used 276 selected genes from the genome of *C. sakazakii* strain BAA-894, the first *Cronobacter* genome sequenced and released in 2008 for interrogation of strains representing six of the seven *Cronobacter* spp. The second study described by Kucerova et al. (28) featured an array that was designed using the entire genome of *C. sakazakii* strain BAA-894 for comparison with strains representing five of the seven *Cronobacter* species. Thus, within the first 2 years of release of the first *Cronobacter* genome, microarrays were being introduced as tools to study the genomic diversity of this genus. Both of these studies used a common method of analysis which incorporated the use of direct comparisons of gene probe intensities obtained for the different species (strains) to probe intensities from similar genes of the reference strain, *C. sakazakii* BAA-894.

In contrast, the microarray used in the present study represents a multi-genome array containing the annotated pan genome of 15 sequenced strains of *Cronobacter*, representative of all seven species. The design of this microarray provides us the opportunity to survey specific genes from different *Cronobacter* species and within each species group, which is a prerequisite of great value to an investigator who may study these closely related species. Moreover, the probe set design provides the opportunity to assess the total gene content of each strain without the need to compare hybridization intensities to a reference genome which previously had been an absolute requirement for accurate comparative genomic hybridization (CGH) studies (28, 29). Thus the number of gene differences is based on strain-to-strain comparisons and “gene difference or divergence” is defined as an eightfold difference in the RMA-summarized probe set intensities for each gene. This is different than the traditional side by side comparison of a fold increase or decrease for each gene of a strain compared to the intensity of that gene contained in the reference strain. However, there is one caveat and that is the genomes used for the design of the microarray were nevertheless used as “controls” to assess the validation of the chip. So, reference strain intensity is not used in the analysis of each gene target directly, but the gene content of reference strains was used to assess the performance (accuracy) of the chip. Recently Brady et al. (23) re-evaluated the taxonomy of the genus *Enterobacter*, and based primarily on multi-locus sequence analysis (MLSA) using partial sequences of four housekeeping genes (*gyrB*, *rpoB*, *infB*, and *atpD*), proposed that *E. helveticus*, *E. pulveris*, and *E. turicensis* be recognized as new species within the genus of *Cronobacter*. These *Enterobacter* species were originally isolated from dried fruit powders, PIF, a number of PIF production environments, and other dried food ingredients by Stephan et al. (30, 31), and were excluded from the genus *Cronobacter* by Iversen et al. (6). At that time, the decision to exclude these *Enterobacter* spp. from the *Cronobacter* genus was based on differences in their phenotypic characteristics, as well as data from DNA–DNA hybridization, and the phylogenetic analysis of the *rpoB* gene (30, 31). However, these novel species do share several phenotypic and metabolic characteristics with *Cronobacter*, such as resistance to desiccation, production of a yellow *Pantoea*-like, carotenoid pigment (9) and constitutive metabolism of 5-bromo-4-chloro-3-indolyl-α-d-glucopyranoside, which is the feature used in the differentiation of presumptive *Cronobacter* colonies growing on the chromogenic and selective *Cronobacter* isolation agars (32) used today. In an effort to further clarify the taxonomic standing of *C. pulveris*, *C. helveticus*, and *C. zurichensis* as proposed by Brady et al. (23), we interrogated strains of these species using the DNA microarray. The results showed that these three species do not belong to the *Cronobacter* genus, a finding that provides further support for the polyphasic taxonomic approach described by Stephan et al. (25) to place these strains into two new genera, *Franconibacter* (*F. helveticus* and *F. pulveris*) and *Siccibacter* (*S. turicensis*).

The comprehensive set of data derived from this study clearly shows that the pan genomic microarray cannot only evaluate the global genomic diversity among the seven species of *Cronobacter*, but it can also discriminate among individual and closely related strains within each species. The latter is an important feature for the newly emerging fields of microbial forensics and molecular epidemiology, where the ability to uniquely classify and differentiate among closely related strains is of unparalleled significance in conducting attribution investigations during foodborne outbreaks (20). Recently the microarray was used by Yan et al. (27) to investigate the genomic diversity of several clinical and environmental strains of *C. sakazakii* which were isolated from the environment of a group of European PIF manufacturing facilities. As described, the results of that study showed that the microarray could separate 25 *C. sakazakii* ST4 strains into two distinct subclades which suggested that there may be two evolutionary lineages associated with ST4 strains. Furthermore, microarray analysis showed that these two lineages differed in a total of 95 unique genes, of which many were phage-related genes (seven related genes) and 17 of these unique genes were associated with the pESA3-encoded type six secretion system (T6SS) gene cluster as described by Franco et al. (13). It was interesting to find that the 25 *C. sakazakii* strains were PCR-positive for *repA* (the origin of plasmid replication and incompatibility class marker (ESA_pESA3, location 115 to 588), which signifies that these strains possess the pESA3 common virulence plasmid. Furthermore, their analysis revealed that most of the strains representing each lineage possessed the 5′ end of the T6SS gene cluster. However, none of the ST4 lineage one strains possessed the *vgrG* gene, while eight of nine strains of ST-4 lineage 2 strains did. Additionally, the lineage-specific pattern was repeated for the 3′ end of the T6SS gene cluster in that lineage one strains were negative for *vgrG*, and the two other regions of this end of the gene cluster. Franco et al. (13) described the T6SS region as a region that is in “great genetic flux.” Could these changes reflect an evolutionary transition which is currently taking place in these strains? Do these changes translate into an increased ability to cause serious illness in neonates? The need to understand and further characterize these pathogens has never been more urgent.

Interestingly, the finding of a gene encoding for a putative virulence factor, the macrophage infectivity potentiator-related protein which is thought to play a role in survival of *Cronobacter* within macrophages suggests that this protein may act as a cationic lysosomotropic agent, such as ammonium chloride, which has been shown to inhibit phagosome acidification and phagosome-lysosome fusion as a similar homolog in *Legionella pneumophila* has been found as described by Engleberg et al. (26). Could the demonstrated species-specificity be related to their ability to cause meningitis? These questions remain unanswered.

Though the primary focus of the research described in this study centered on using a pan genome-based DNA microarray to subtype *Cronobacter* strains and to understand divergence of species-specific alleles, the microarray will also be just as useful in understanding gene expression or transcriptomics, i.e., understanding the physiology of an organism grown under differing growth conditions. For example, using transcriptomics in conjunction with RNA sequencing should provide researchers valuable insights into the survival strategies used by *Cronobacter* in PIF. Additional uses of the microarray would be the interrogation of additional strains within the clinical community which will help confirm or identify relevant virulence factors. Lastly, the corroborating data from the microarray and the alignment of metadata associated with the strains under investigation has led us to conclude that the microarray will be an undeniably powerful tool in the future of food safety and public health against the threat of foodborne illness epidemics caused by *Cronobacter*.

## Conflict of Interest Statement

The authors declare that the research was conducted in the absence of any commercial or financial relationships that could be construed as a potential conflict of interest.

## Supplementary Material

The Supplementary Material for this article can be found online at http://journal.frontiersin.org/article/10.3389/fped.2015.00036
