# Supplementary material for: Development of a Custom-Designed, Pan Genomic DNA Microarray to Characterize Strain-Level Diversity among Cronobacter spp
Source: Front Pediatr. 2015 Apr 30;3:36. doi: 10.3389/fped.2015.00036 (PMC4415424; doi:10.3389/fped.2015.00036)
Supplement: Supplementary file 1 [file Table_1.PDF]

Supplemental Table 1. List of strains used in this study

Strain	Isolation Date	Genus Species	Source	Country	Serotype	Sequence type
Ccon LMG 26250	2006-2009	*condimenti*	Food, spiced sausage	Slovakia	ND	98
Cdub CFS237	2004-00	*dublinensis*	Environmental, Milk powder production facility	Ireland	Cdub O:1	106
Cdub E187_2		*dublinensis*	Environmental, Milk powder production facility	Ireland	Cdub O:1	106
Cdub 0743−75	1975-00	*dublinensis*	Clinical, Foot wound	USA	Cdub O:1	41
Cdub 3−27b	2011-00	*dublinensis*	Food, Nuts	Korea	Cdub O:1	ND
Cdub1108−29−3	8/29/2011	*dublinensis*	Food, Vegetable	Thailand	Cdub O:1	ND
Cdub1028		*dublinensis*	Environment, Milk power plant	Phillipines	Cdub O:1	ND
Cdub1027		*dublinensis*	Environment, Milk power plant	Phillipines	ND	ND
Cdub 853057	4/11/2014	*dublinensis*	Fresh Basil	USA	ND	ND
Cdub 1105−6	5/6/2011	*dublinensis*	Food,Vegetable	Thailand	ND	ND
Cdub 5960−70	1970-00	*dublinensis*	Clinical, Blood	USA	ND	5
Cdub E791		*dublinensis*	Unknown	Ireland	Cdub O:1	ND
Cdub 6−3Gb	2011-00	*dublinensis*	Food, Nuts	Korea	Cdub O:1	ND
Cdub Sun−1	2009-00	*dublinensis*	Food,Taro	Korea	ND	ND
Cdub 464	2003-00	*dublinensis*	Environmental, Milk powder production facility	Zimbabwe	Cdub O:1	79
Cdub E515C	2004-00	*dublinensis*	Environmental, water fountain	Switzerland	Cdub O:2	80
Cdub 9079−75B	1975-00	*dublinensis*	Unknown	USA	Cdub O:2	36
CdubE798		*dublinensis*	Unknown	Ireland	Cdub O:2	ND
CdubE799_2		*dublinensis*	Unknown	Ireland	Cdub O:2	ND
Cdub6−15Gb	2011-00	*dublinensis*	Food, Nuts	Korea	Cdub O:2	ND
CdubORA114244	1/14/2014	*dublinensis*	Oregano	USA	Cdub O:2	ND
Cmuy 456B		*muytjensii*	Unknown	Unknown	Cmuy O:2	ND
Cmuy 51329B		*muytjensii*	Unknown	USA	Cmuy O:2	81
Cmuy J77		*muytjensii*	Food, Anise, Spice	Jordan	Cmuy O:2	ND
Cmuy J171		*muytjensii*	Fennel, food, spice	Jordan	ND	ND
Cmuy 3523−75	1975-00	*muytjensii*	Clinical, Bone marrow	USA	Cmuy O:2	5
Cmuy J174		*muytjensii*	Food, Anise, Spice	Jordan	Cmuy O:1	ND
Cmuy J95		*muytjensii*	Food, Anise, Spice	Jordan	ND	ND
Cmuy E888		*muytjensii*	Food, Milk Powder	Ireland	ND	ND
Cmuy J112		*muytjensii*	Food, Liquorice	Jordan	ND	ND
Cmuy E769B		*muytjensii*	Food, Milk powder	Ireland	Cmuy O:1	ND
CmuyJ176		*muytjensii*	Food, Thyme, Spice	Jordan	ND	ND
CmalCI695		*malonaticus*	Food, Infant food	Switzerland	Cmal O:2	ND
CmalCI825_5ug		*malonaticus*	Clinical	USA	Cmal O:2	7
CmalMd25g	2012-00	*malonaticus*	Fly, *Musca domestica* , gut	USA	Cmal O:2	ND
Cmal2149	2004-00	*malonaticus*	Clinical, CSF	USA	Cmal O:2	ND
Cmal2153	2004-00	*malonaticus*	Clinical, Blood	USA	Cmal O:2	ND
Cmal0939A−75	1975-00	*malonaticus*	Clinical, Nose	USA	Cmal O:2	ND
CmalCI765		*malonaticus*	Clinical	Switzerland	Cmal O:2	ND
CmalE763		*malonaticus*	Clinical	Ireland	Cmal O:2	ND
CmalE608		*malonaticus*	Clinical	Ireland	Cmal O:2	ND
CmalE618		*malonaticus*	Clinical	Ireland	Cmal O:2	ND
CmallCI868		*malonaticus*	Clinical	Switzerland	Cmal O:2	ND
CmalE685		*malonaticus*	Food	USA	Cmal O:2	ND
CmalE754		*malonaticus*	Clinical	Switzerland	Cmal O:2	ND
CmalLR577		*malonaticus*	Environmental	USA	Cmal O:2	ND
CmalE766		*malonaticus*	Food, Milk powder	Ireland	Cmal O:2	ND
CmalE265		*malonaticus*	Food, Milk powder	Switzerland	Cmal O:2	ND
CmalLR580		*malonaticus*	Environmental	USA	Cmal O:2	ND
CmalJ160		*malonaticus*	Environmental,Vacuum dust	Jordan	Cmal O:2	ND
CmalJ168BB		*malonaticus*	Food, Spices	Jordan	Cmal O:2	ND
CmalLR686C	12/1/2005	*malonaticus*	Food, PIF ingredient	USA	Cmal O:1	ND
CmalE684		*malonaticus*	Food	Ireland	Cmal O:1	ND
CmalE831		*malonaticus*	Clinical	Ireland	Cmal O:1	ND
CmalE833		*malonaticus*	Clinical	Ireland	Cmal O:1	ND
CmalE615		*malonaticus*	Clinical	Ireland	Cmal O:1	ND
CmalE831_2		*malonaticus*	Clinical	Ireland	Cmal O:1	ND
CmalMd99g	2012-00	*malonaticus*	Fly, *Musca domestica* , gut	Ireland	Cmal O:1	ND
CmalSHSM3hTF	8/1/2009	*malonaticus*	Environmental, Tomato field	USA	Cmal O:1	ND
CsakNM1241	2008-00	*sakazakii*	Clincal, CSF	USA	Csak O:4	ND
CsakBaa894dcpa		*sakazakii*	*Cronobacter* plasminogen activator deficient mutant	USA	Csak O:1	ND
CsakBAA-894	4/1/2001	*sakazakii*	Food, PIF	USA	Csak O:1	1
CsakBaa894dpESA3	12/12/2010	*sakazakii*	Plasmid pESA3 cured derivative	USA	Csak O:1	ND
CsakLR737	6/1/2007	*sakazakii*	Food, Org Hi PDI Flour	USA	Csak O:3	ND
Csak3−21	2011-00	*sakazakii*	Food, Nuts	Korea	Csak O:1	ND
CsakCF684	1/22/2013	*sakazakii*	Chicken Feed	USA	Csak O:1	ND
CsakE756		*sakazakii*	Clinical	Ireland	Csak O:4	ND
CsakEs35	2002-00	*sakazakii*	Clinical	Israel	Csak O:1	ND
CsakO#23mBOregano	4/7/204	*sakazakii*	Food, Oregano, Spice	USA	Csak O:1	ND
Csak29544_5ug	1978-00	*sakazakii*	Clinical, Child, Throat swab	USA	Csak O:1	8
CsakMd40g_2	2012-00	*sakazakii*	Fly, *Musca domestica* , gut	USA	Csak O:2	ND
CsakE899_5ug	1981-00	*sakazakii*	Clinical	USA	Csak O:2	4
Csak4.01C_5ug		*sakazakii*	Food, PIF	USA	Csak O:2	ND
CsakNM1240	2008-00	*sakazakii*	Clinical, Blood	USA	Csak O:4	4
CsakNM1242	2008-00	*sakazakii*	Clinical, Brain, matches NM1240	USA	Csak O:4	4
Csak20060625	2006-00	*sakazakii*	Clinical	USA	Csak O:2	ND
Csak2151_5ug	2003-00	*sakazakii*	Clinical, CSF	USA	Csak O:2	4
Csak936975	1975-00	*sakazakii*	Unknown	USA	Csak O:2	4
Csak9368−75	1975-00	*sakazakii*	Unknown	USA	Csak O:2	ND
Csak255N		*sakazakii*	Clinical	Ireland	Csak O:2	4
Csak206N		*sakazakii*	Clinical	Ireland	Csak O:2	4
Csak306N		*sakazakii*	Clinical	Ireland	Csak O:2	4
CsakLR579		*sakazakii*	Environmental	USA	Csak O:3	ND
Csak2156−3	2005-00	*sakazakii*	Clinical, Blood	USA	Csak O:3	ND
CsakLR746	10/1/2007	*sakazakii*	Food, ISO III powder	USA	Csak O:3	ND
CsakMd70g	2012-00	*sakazakii*	Fly, *Musca domestica* , gut	USA	Csak O:2	ND
CsakJ178		*sakazakii*	Food, Chamomile, Spice	Jordan	Csak O:3	ND
CsakJ96		*sakazakii*	Food, Fennel, Spice	Jordan	Csak O:3	ND
CsakMd6g	2012-00	*sakazakii*	Fly, *Musca domestica* , gut	USA	Csak O:3	ND
CsakSP291_5ug		*sakazakii*	Environment, PIF powder production facility	Ireland	Csak O:2	4
CsakE837		*sakazakii*	Clinical	Ireland	Csak O:2	ND
Csak777122	10/10/2012	*sakazakii*	Food, Hulled Seseme Seed	India	Csak O:4	ND
CsakCI764_5ug		*sakazakii*	Clinical	Switzerland	Csak O:4	ND
CsakJ204_2		*sakazakii*	Food, Liquorice	Jordan	Csak O:7	ND
CsakLR582		*sakazakii*	Environmental	USA	Csak O:4	ND
CsakLR643	2/22/2004	*sakazakii*	Food, Soy flour	USA	Csak O:3	ND
CsakLR646	2/22/2005	*sakazakii*	Food, Soy flour	USA	Csak O:1	ND
Csak1−15	2011-00	*sakazakii*	Food, Nuts	Korea	Csak O:2	ND
Csak5−21G	2011-00	*sakazakii*	Food, Nuts	Korea	Csak O:2	ND
CsakFCHB8142014	4/4/2014	*sakazakii*	Chicken feed course	USA	Csak O:4	ND
Csak760029	2/7/2013	*sakazakii*	Food, Whole grain, Corn	Puerto Rico	Csak O:4	ND
CsakKW3	2009-00	*sakazakii*	Food, Dried Hot Pepper	Korea	Csak O:4	ND
Csak788569	12/21/2012	*sakazakii*	Food, Siberian Ginseng, Eleutherom Root Powder (*Eleutherococcus sentiocosus*)	China	Csak O:2	ND
CsakKW9	2009-00	*sakazakii*	Food, Sorghum	Korea	ND	ND
CsakEs718		*sakazakii*	Clinical	USA	Csak O:3	ND
CsakJ32		*sakazakii*	Food, Baby food	Jordan	Csak O:3	ND
CsakKW1	2009-00	*sakazakii*	Food, Barely	Korea	ND	ND
CsakLR642	2/12/2005	*sakazakii*	Food, Sodium casinate	USA	Csak O:3	ND
Cuni680		*universalis*	Food	Switzerland	ND	ND
Cuni797−2	1954-00	*universalis*	Water	Uk	Cuni O:1	54
CturE681−2		*turicensis*	Food, Infant food	Switzerland	Ctur O:3	ND
CturE625B		*turicensis*	Food, Infant food	Switzerland	Ctur O:3	ND
CturSh41s	2012-00	*turicensis*	Fly, *Sarcophaga haemorrhoidalis* , surface	USA	ND	ND
Ctur676		*turicensis*	Food	Switzerland	Ctur O:3	ND
CturE626		*turicensis*	Food, Infant food	Switzerland	Ctur O:3	ND
Ctur688		*turicensis*	Food	Switzerland	ND	ND
Ctur3032	2005-00	*turicensis*	Clinical, Blood	Switzerland	Ctur O:1	19
CturE866		*turicensis*	Clinical, Blood	Ireland	Ctur O:1	19
SalSTM LT2 (ATCC 700720)		*S. typhimurium*	FDA Culture collection	Unknown	ND	ND
Stur508 (LMG 23730^{T})		*S. turicensis*	Fruit powder	Switzerland	ND	ND
Fhelz1159 (LMG 23733)		*F. helveticus*	Fruit powder	Switzerland	ND	ND
Fhelz513 (LMG 23732^{T})		*F. helveticus*	Fruit powder	Switzerland	ND	ND
Fpul1160 (LMG 24058)		*F. pulveris*	Apple fruit powder	Switzerland	ND	ND
Fpul601 (LMG 24057^{T})		*F. pulveris*	Apple fruit powder	Switzerland	ND	ND
Cfreundii576		*C. freundii*	FDA Culture collection	Unknown	ND	ND
Kpneumoniae214		*K. pneumoniae*	FDA Culture collection	Unknown	ND	ND
