# Supplementary material for: Development of a Custom-Designed, Pan Genomic DNA Microarray to Characterize Strain-Level Diversity among Cronobacter spp
Source: Front Pediatr. 2015 Apr 30;3:36. doi: 10.3389/fped.2015.00036 (PMC4415424; doi:10.3389/fped.2015.00036)
Supplement: Supplementary file 4 [file Table_4.PDF]

Supplemental Table S4. Microarray probe sets, probe set % similarities, NCBI protein and gene annotations and genome regions, corresponding to the malonate and *myo* -Inositol gene clusters of the *C. dublinensis* strains.

ProbeSet	Probe species ID	ProbeSet	% similarity	NCBI protein	NCBI annotations	GR	CdubCFS237	CdubE187_2	Cdub3_27b	Cdub0743_75	Cdub1108_29_3	Cdublac6_3Gb	Cdub1027	Cdub1028	Cdub1105_6	Cdub5960_70	CdubE791	CdubSun_1	853057Cdub	CdubE515C	Cdub6_15Gb	Cdub9079_75B	CdubE798	CdubE799_2	CdubORA114244
413499.3.2827	Cdublac	2827	35.38	ABU75697	Major myo-inositol transporter IolT	GR29 - myo inositol	P	P	P	P	P	P	P	P	P	P	P	P	P	A	A	A	A	A	A
413499.3.2828	Cdublac	2828			hypothetical protein	GR29 - myo inositol	A	A	P	A	A	P	P	A	A	A	A	A	A	A	A	A	A	A	A
413499.3.2829	Cdublac	2829	85.28	AEW59229	Inosose dehydratase (EC 4.2.1.44)	GR29 - myo inositol	P	P	P	P	P	P	P	P	P	P	P	P	P	A	A	A	A	A	A
413499.3.2830	Cdublac	2830	89.4	AEW59228	Glyceraldehyde-3-phosphate ketol-isomerase (EC 5.3.1.1)	GR29 - myo inositol	P	P	P	P	P	P	P	P	P	P	P	P	P	A	A	A	A	A	A
413499.3.2831	Cdublac	2831	85.16	AEW59227	Myo-inositol 2-dehydrogenase 1 (EC 1.1.1.18)	GR29 - myo inositol	P	P	P	P	P	P	P	P	P	P	P	P	P	A	A	A	A	A	A
413499.3.2832	Cdublac	2832	26.68	ABU78980	Epi-inositol hydrolase (EC 3.7.1.-)	GR29 - myo inositol	P	P	P	P	P	P	P	P	P	P	P	P	P	A	A	A	A	A	A
413499.3.2833	Cdublac	2833			hypothetical protein	GR29 - myo inositol	P	P	P	P	P	P	P	P	P	P	P	P	P	A	A	A	A	A	A
413499.3.2834	Cdublac	2834	27.93	ABU79358	5-keto-2-deoxygluconokinase (EC 2.7.1.92) / uncharacterized domain	GR29 - myo inositol	P	P	P	P	P	P	P	P	P	P	P	P	P	A	A	A	A	A	A
413499.3.2835	Cdublac	2835	22.38	ABU75818	Predicted transcriptional regulator of the myo-inositol catabolic operon	GR29 - myo inositol	P	P	P	P	P	P	P	P	P	P	P	P	P	A	A	A	A	A	A
413499.3.2836	Cdublac	2836	85.45	ACI10093	5-deoxy-glucuronate isomerase (EC 5.3.1.-)	GR29 - myo inositol	P*	P*	P*	P	P	P	P	P	P	P	P	P	P	A	A	A	A	A	A
413499.3.2837	Cdublac	2837	31.36	ABU77299	Methylmalonate-semialdehyde dehydrogenase [inositol] (EC 1.2.1.27)	GR29 - myo inositol	P	P	P	P	P	P	P	P	P	P	P	P	P	A	A	A	A	A	A
413499.3.2838	Cdublac	2838	75.46	AEW59220	Inosose isomerase (EC 5.3.99.-)	GR29 - myo inositol	P	P	P	P	P	P	P	P	P	P	P	P	P	A	A	A	A	A	A
413498.3.1473	Cdubdub	1473	23.77	ABU77068	LysR family transcriptional regulator YneJ	GR34 - malonate	P	P	P	P	P	A	A	A	A	A	A	A	A	A	A	A	A	A	A
413498.3.1474	Cdubdub	1474	35.66	ABU77502	Malonyl CoA-acyl carrier protein transacylase;Ontology_term=KEGG_ENZYME:2.3.1.39	GR34 - malonate	P	P	P	P	P	A	A	A	A	A	A	A	A	A	A	A	A	A	A
413498.3.1475	Cdubdub	1475	63.9	AEW61164	phosphoribosyl-dephospho-CoA_transferase	GR34 - malonate	P	P	P	P	P	A	A	A	A	A	A	A	A	A	A	A	A	A	A
413498.3.1476	Cdubdub	1476	87.77	ACI09216	malonate_transporter_MdcF;mdcF	GR34 - malonate	P	P	P	P	P	A	A	A	A	A	A	A	A	A	A	A	A	A	A
413498.3.1477	Cdubdub	1477	81.95	AEW61166	MdcE	GR34 - malonate	P	P	P	P	P	A	A	A	A	A	A	A	A	A	A	A	A	A	A
413498.3.1478	Cdubdub	1478	39.13	ABU76170	Acetyl-coenzyme A carboxyl transferase beta chain;Ontology_term=KEGG_ENZYME:6.4.1.2	GR34 - malonate	P	P	P	P	P	A	A	A	A	A	A	A	A	A	A	A	A	A	A
413498.3.1479	Cdubdub	1479	80.56	ACI11832	"malonate_decarboxylase	GR34 - malonate	P	P	P	P	P	A	A	A	A	A	A	A	A	A	A	A	A	A	A
413498.3.1480	Cdubdub	1480	33.85	ABU75626	2-(5''-triphosphoribosyl)-3'-dephosphocoenzyme-A synthase;Ontology_term=KEGG_ENZYME:2.7.8.25	GR34 - malonate	P	P	P	P	P	A	A	A	A	A	A	A	A	A	A	A	A	A	A
413498.3.1481	Cdubdub	1481	93.28	ACI08064	"malonate_decarboxylase	GR34 - malonate	P	P	P	P	P	A	A	A	A	A	A	A	A	A	A	A	A	A	A
						C. dublinensis ssp. identity	*dublinensis*	*dublinensis*	*dublinensis*	*dublinensis*	*dublinensis*	*lactaridi*	*lactaridi*	*lactaridi*	*lactaridi*	*lactaridi*	*lactaridi*	*lactaridi*	*lactaridi*	*lausannensis*	*lausannensis*	*lausannensis*	*lausannensis*	*lausannensis*	*lausannensis*

Abbreviations: Presence absence calls are pased on probe set fluorescent intenstities: P=12, P*=11-12, A=<11; Orange highlights and Gray highlights mean positive and negative hybridization results respectively for each RMA-deirved result.
