# Supplementary figures and images for: Development of a Custom-Designed, Pan Genomic DNA Microarray to Characterize Strain-Level Diversity among Cronobacter spp
Source: Front Pediatr. 2015 Apr 30;3:36. doi: 10.3389/fped.2015.00036 (PMC4415424; doi:10.3389/fped.2015.00036)

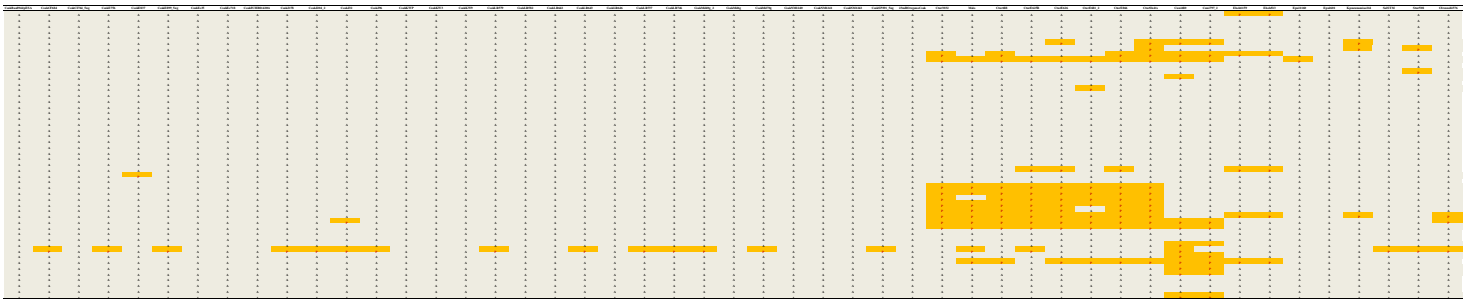

Supplement: Supplementary file 5 [file Table_5.PDF]
