# Supplementary material for: Development of a Custom-Designed, Pan Genomic DNA Microarray to Characterize Strain-Level Diversity among Cronobacter spp
Source: Front Pediatr. 2015 Apr 30;3:36. doi: 10.3389/fped.2015.00036 (PMC4415424; doi:10.3389/fped.2015.00036)
Supplement: Supplementary file 7 [file Table_7.PDF]

Supplemental Table S7. Microarray probe sets, probe set % similarities, NCBI protein and gene annotations, corresponding gene loci, and hybridization results which correspond to the Macrophage infectivity potentiator-related protein genes of *C. turicensis, C. malonaticus, C. muytjensii, and C. sakazakii*.

ProbeSet	Probe species ID	% similarity	NCBI protein	NCBI annotations	BAA loci
6666.1845.2266	Ctur z3032.2266	94.38	ABU77122	Macrophage infectivity potentiator-related protein	ESA_01868
413503.3.254	Cmal 23826.254	97.19	ABU77122	Macrophage infectivity potentiator-related protein	ESA_01868
413501.3.2272	Cmuy 51329.2272	93.26	ABU77122	Macrophage infectivity potentiator-related protein	ESA_01868
6666.1844.1726	Csak BAA894.1726	100	ABU77122	Macrophage infectivity potentiator-related protein	ESA_01868

Strains analyzed

ProbeSet	353057Cdub	Cdub3_27b	Cdub0743_75	Cdub1027	Cdub1028	Cdub1105_6	Cdub1108_29_3	Cdub5960_70	Cdub6_15Gb	Cdub6_3Gb	Cdub9079_75B	CdubCFS237	CdubE187_2	CdubE515C	CdubE791	CdubE798	CdubE799_2	CdubORA114244	CdubSun_1
6666.1845.2266	A	A	A	A	A	A	A	A	A	A	A	A	A	A	A	A	A	A	A
413503.3.254	A	A	A	A	A	A	A	A	A	A	A	A	A	A	A	A	A	A	A
413501.3.2272	A	A	A	A	A	A	A	A	A	A	A	A	A	A	A	A	A	A	A
6666.1844.1726	A	A	A	A	A	A	A	A	A	A	A	A	A	A	A	A	A	A	A

ProbeSet	Cmal0939A_75	Cmal2149	Cmal2153	CmalCI696	CmalCI765	CmalCI825_5ug	CmalE265	CmalE608	CmalE615	CmalE618	CmalE684	CmalE685	CmalE754	CmalE763	CmalE766	CmalE831	CmalE831_2	CmalE833	CmalJ160	CmalJ168BB	CmalCI868	CmalLR577	CmalLR580	CmalLR686C	CmalMd25g	CmalMd99g	CmalSHSM3hTF
6666.1845.2266	A	A	A	A	A	A	A	A	A	A	A	A	A	A	A	A	A	A	A	A	A	A	A	A	A	A	A
413503.3.254	P	P	P	P	P	P	P	P	P	P	P	P	P	P	P	P	P	P	P	P	P	P	P	P	P	P	P
413501.3.2272	A	A	A	A	A	A	A	A	A	A	A	A	A	A	A	A	A	A	A	A	A	A	A	A	A	A	A
6666.1844.1726	A	A	A	A	A	A	A	A	A	A	A	A	A	A	A	A	A	A	A	A	A	A	A	A	A	A	A

ProbeSet	Cmuy3523_75	Cmuy4568	Cmuy51329B	CmuyE769B	CmuyE888	CmuyJ112	CmuyJ171	CmuyJ174	CmuyJ176	CmuyJ77	CmuyJ95
6666.1845.2266	A	A	A	A	A	A	A	A	A	A	A
413503.3.254	A	A	A	A	A	A	A	A	A	A	A
413501.3.2272	P	P	P	P	P	P	P	P	P	P	P
6666.1844.1726	A	A	A	A	A	A	A	A	A	A	A

ProbeSet	Csak1_15	Csak2060625	Csak2151_5ug	Csak2156_3	Csak29544_5ug	Csak3_21	Csak4_01C_5ug	Csak5_21G	Csak760029	Csak777122	Csak788569	Csak9368_75	Csak936975	CsakBAA_10ug	CsakBAA_5ug	CsakBAA894_2ug	CsakBaa894dpESA3	206NCsak	255NCsak	306NCsak	CsakCF684	CsakCI764_5ug	CsakE756	CsakE837	CsakE899_5ug	CsakEs35
6666.1845.2266	A	A	A	A	A	A	A	A	A	A	A	A	A	A	A	A	A	A	A	A	A	A	A	A	A	A
413503.3.254	A	A	A	A	A	A	A	A	A	A	A	A	A	A	A	A	A	A	A	A	A	A	A	A	A	A
413501.3.2272	A	A	A	A	A	A	A	A	A	A	A	A	A	A	A	A	A	A	A	A	A	A	A	A	A	A
6666.1844.1726	P	P	P	P	P	P	P	P	P	P	P	P	P	P	P	P	P	P	P	P	P	P	P	P	P	P

ProbeSet	CsakEs718	CsakFCHBB142014	CsakJ178	CsakJ204_2	CsakJ32	CsakJ96	CsakKW1	CsakKW3	CsakKW9	CsakLR579	CsakLR582	CsakLR642	CsakLR643	CsakLR646	CsakLR737	CsakLR746	CsakMd40g_2	CsakMd6g	CsakMd70g	CsakNM1240	CsakNM1241	CsakNM1242	CsakSP291_5ug	23mBOreganoCsak
6666.1845.2266	A	A	A	A	A	A	A	A	A	A	A	A	A	A	A	A	A	A	A	A	A	A	A	A
413503.3.254	A	A	A	A	A	A	A	A	A	A	A	A	A	A	A	A	A	A	A	A	A	A	A	A
413501.3.2272	A	A	A	A	A	A	A	A	A	A	A	A	A	A	A	A	A	A	A	A	A	A	A	A
6666.1844.1726	P	P	P	P	P	P	P	P	P	P	P	P	P	P	P	P	P	P	P	P	P	P	P	P

ProbeSet	Ctur3032	Ctur676	Ctur688	CturE625B	CturE626	CturE681_2	CturE866	CturSh41s	Cuni680	Cuni797_2	ConLMG26250	Ehelv1159	Ehelv513	Epull1160	Epul601	Kpneumoniae214	Cfreundii676	SalSTM	Stur308
6666.1845.2266	P	P	P	P	P	P	P	P	A	A	A	A	A	A	A	A	A	A	A
413503.3.254	A	A	A	A	A	A	A	A	A	A	A	A	A	A	A	A	A	A	A
413501.3.2272	A	A	A	A	A	A	A	A	A	A	A	A	A	A	A	A	A	A	A
6666.1844.1726	A	A	A	A	A	A	A	A	A	A	A	A	A	A	A	A	A	A	A

Highlighted data points: Gray, refers to gene absent; orange, refers to gene present.
